# The genetic architecture of gene expression regulation in a *Citrus x Poncirus* hybrid tolerant to Huanglongbing

**DOI:** 10.3389/fpls.2025.1627531

**Published:** 2025-09-04

**Authors:** Isaac A. Diaz, Omar Zayed, Emmanuel Ávila De Dios, Claire Jiang, Kim D. Bowman, Danelle K. Seymour

**Affiliations:** ^1^ Department of Botany and Plant Sciences, University of California, Riverside, Riverside, CA, United States; ^2^ U.S. Horticultural Research Laboratory, Agricultural Research Service, United States Department of Agriculture, Ft. Pierce, FL, United States

**Keywords:** *Citrus*, *Poncirus*, Huanglongbing, allele-specific expression (ASE), cis-regulation

## Abstract

Interspecific hybridization is a common and effective strategy for producing disease resilient citrus cultivars, including those with tolerance to Huanglongbing (HLB) disease. Several HLB-tolerant cultivars have been developed through hybridization of mandarins (*Citrus reticulata*) with their wild relative *Poncirus trifoliata*. One such cultivar, ‘US-897’, exhibits robust tolerance to the bacteria causing HLB disease, *Candidatus Liberibacter asiaticus* (*C*Las). To explore the genetic architecture of the early transcriptional response to *Candidatus* Liberibacter asiaticus (*C*Las) infection in ‘US-897’, we performed transcriptomic analysis of the hybrid and its parents, ‘Cleopatra’ (*C. reticulata*) and ‘Flying Dragon’ (*P. trifoliata*). A haplotype-resolved genome for ‘US-897’ was generated using PacBio HiFi sequencing reads to support quantification of the expression of both the *Citrus and Poncirus* alleles. By profiling gene expression in this parent-offspring trio, we were able to determine the mode of inheritance for genes differentially expressed between parents (‘Cleopatra’ and ‘Flying Dragon’) and their interspecific hybrid (‘US-897’), with the majority genes exhibiting non-additive patterns of gene expression inheritance. Additionally, analysis of allele-specific expression in the hybrid ‘US-897’ revealed the contribution of cis- versus trans-acting regulatory variants on genes with additive and non-additive modes of inheritance. A strong correlation between differential expression between parents and allele-specific expression in ‘US-897’ suggests that cis-regulatory variation is a significant source of expression divergence between species. Finally, genes responsive to infection with *C*Las were identified to explore how gene regulation associated with tolerance to HLB was rewired between *Citrus* and its relative *Poncirus*.

## Introduction

Interspecific hybridization can improve disease resilience by introducing beneficial alleles from the relatives of cultivated crop species. In long-lived perennial tree crops, like those in the genus *Citrus*, interspecific hybridization has led to the development of many commercial cultivars with enhanced disease resistance and improved productivity in disease endemic regions. A current major challenge to citrus production is the bacterial disease Huanglongbing (HLB). The most common cause of HLB in the United States is the phloem-restricted bacterial species *Candidatus* Liberibacter asiaticus (*C*Las) which infects all commercial citrus types and has major impacts on fruit production (Bové, 2006). *C*Las is vectored by the Asian citrus psyllid (*Diaphorina citri*), which first arrived in Florida in 1998 (Hall, 2008). HLB has devastated the citrus industry in Florida (Court and Rahmani, 2017) and genetic solutions, including the development of disease resilient cultivars, are one of the many tools being used to combat HLB disease (Albrecht and Bowman, 2012a; Bowman and McCollum, 2015; Albrecht et al., 2020; Bowman and Albrecht, 2020; Peng et al., 2020).

*Poncirus trifoliata* is a relative of species in the genus *Citrus* with tolerance to HLB (Albrecht and Bowman, 2012a). Several *Citrus* x *Poncirus* hybrids have been shown to have levels of tolerance to *C*Las similar to *P. trifoliata* (Albrecht and Bowman, 2011, 2012a; Bowman and McCollum, 2015), including reduced bacterial titers and limited development of disease symptoms. One hybrid, ‘US-897’, developed from a cross between ‘Cleopatra’ mandarin (*C. reticulata*) and ‘Flying Dragon’, (*P. trifoliata*) exhibits robust tolerance to *C*Las infection as an ungrafted tree (Albrecht and Bowman, 2011). Although the hybrid ‘US-897’ can still be infected by the bacterium, it only exhibits subtle symptoms of infection in both greenhouse studies and field evaluations (Albrecht and Bowman, 2011). In contrast, *C*Las titer rapidly increases in ‘Cleopatra’, the susceptible maternal parent of ‘US-897’, after infection and associated disease symptoms include chlorosis, blotchy mottling of leaves, and reduced overall size. The strong resilience of ‘US-897’ to *C*Las provides an opportunity to characterize the basis of HLB tolerance in an interspecific hybrid.

Improved hybrid performance can result from genetic interactions between parental genomes, including those that may alter patterns of gene expression (Chen, 2013). In the analysis of parent-offspring trios, gene expression can be classified into additive effects, where hybrid expression is intermediate between the two parents, and non-additive effects such as expression-level dominance and transgressive expression including over- and under-dominance (Rapp et al., 2009). There has been a long debate over the genetic architecture underlying hybrid traits (Schwartz and Laughner, 1969; Semel et al., 2006; Krieger et al., 2010; Seymour et al., 2016), and the relative contribution of loci with dominant (Hua et al., 2003; Garcia et al., 2008; Xiao et al., 2021) or overdominant (Rédei, 1962; Vrebalov et al., 2002; Krieger et al., 2010; Guo et al., 2014) contributions to hybrid vigor (Paril et al., 2024). Examples of loci with dominant and overdominant effects on hybrid vigor have been identified across many plant species (Semel et al., 2006; Li et al., 2008; Hashimoto et al., 2021). Overall, the contribution of dominant and overdominant loci to hybrid vigor seems to vary by species and mating system (Huang et al., 2016; Tian et al., 2019; Hashimoto et al., 2021; Xiao et al., 2021). Ultimately, the genetic architecture and patterns of inheritance are important for understanding the regulatory mechanisms underlying hybrid phenotypes, including gene expression.

The genetic architecture of gene regulatory variation in hybrids includes whether genes are cis- or trans-regulated and can provide insight into the mechanistic basis of hybrid phenotypes (Rockman and Kruglyak, 2006). Cis-regulatory variants alter expression of a gene by disrupting regulatory sequences in close proximity to that gene and may include genetic variation in promoters and enhancers. In contrast, trans-regulatory variants perturb the expression of a gene by altering the activity of regulatory molecules, such as transcription factors and regulatory RNAs (Hill et al., 2021). Unlike cis-regulatory variants, trans-regulatory variants may not be located in close physical proximity to their target. Profiling gene expression in parent-offspring trios is commonly used to separate cis- and trans-regulatory effects on expression because both parental alleles, and any associated cis-regulatory variants, experience a shared trans-regulatory environment (Wittkopp et al., 2004; Goncalves et al., 2012; Pereira and Kuzmin, 2024). Cis-regulated genes will exhibit allele-specific expression (ASE) in the hybrid, while trans-regulatory variants will alter the expression of both alleles concordantly (Hill et al., 2021). Dissecting the genetic architecture of gene expression can connect expression divergence to phenotypic divergence and can provide valuable insight into the mechanisms underlying hybrid phenotypes.

Here, we assembled a high-quality, haplotype-resolved reference genome for the HLB-tolerant hybrid ‘US-897’ and performed transcriptomic analyses of the hybrid and its parents, ‘Cleopatra’ (*C. reticulata*) and ‘Flying Dragon’ (*P. trifoliata*), to investigate the genetic architecture of gene expression regulation, including in response to *C*Las infection. Haplotype phasing of the ‘Cleopatra’ and ‘Flying Dragon’ chromosomes in the diploid genome of ‘US-897’ enabled quantification of allele-specific expression (ASE) through accurate assignment of RNA-sequencing reads to either the *Citrus* or *Poncirus* haplotype, which is superior to relying on a haploid reference genome (Wu et al., 2023). By profiling gene expression in this parent-offspring trio, we were able to determine the mode of inheritance for genes differentially expressed between parents (‘Cleopatra’ and ‘Flying Dragon’) and their interspecific hybrid (‘US-897’). Additionally, analysis of allele-specific expression in the hybrid ‘US-897’ revealed the contribution of cis- versus trans-acting regulatory variants on genes with additive, dominant, and transgressive inheritance. Finally, genes responsive to infection with *C*Las were identified to explore the regulatory architecture of pathogen response.

## Results

### Assembly of a diploid reference genome of F_1_ hybrid ‘US-897’

**Figure 1:**
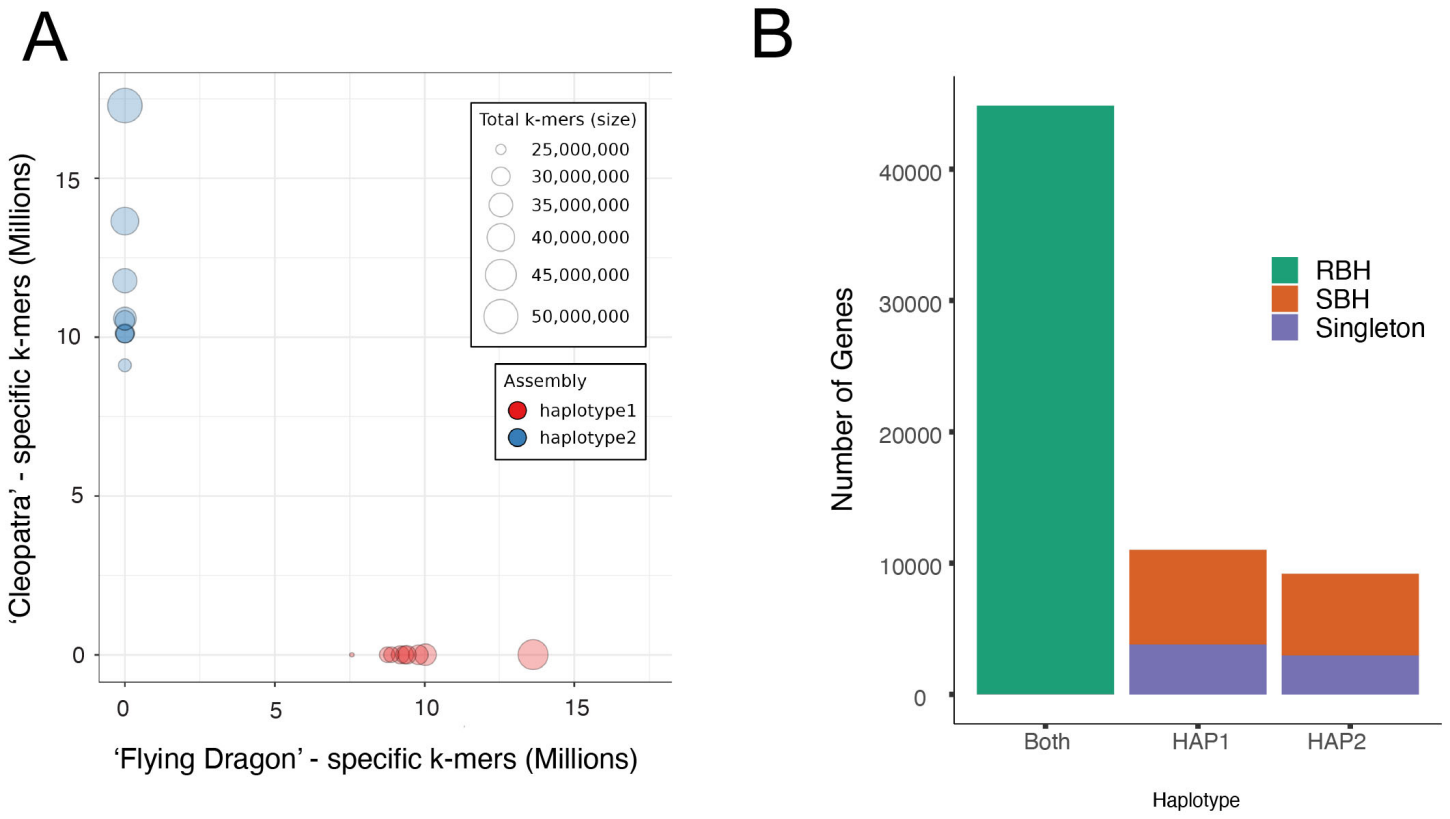
A haplotype-resolved assembly of the interspecific hybrid ‘US-897’. **(A)** The abundance of parent-specific “hap-mers” (k=19) in each haplotype of the ‘US-897’ diploid reference genome. Hap-mers were identified from ‘Cleopatra’ and ‘Flying Dragon’ whole-genome sequencing reads (Wu et al., 2018; Peng et al., 2020). **(B)** Classification of genes based on their sequence homology and collinearity between *Citrus* and *Poncirus* haplotypes of ‘US-897’. Reciprocal best hits (RBH) are allelic between haplotypes, single best hits (SBH) are orthologous sequences that are not reciprocal best hits, and singletons are genes that are unique to a haplotype.

To quantify the expression of both the *Citrus* and *Poncirus* alleles in the interspecific hybrid, a haplotype-resolved chromosome-level genome of ‘US-897’ was assembled from 65.57 Gb of PacBio HiFi sequencing reads (108.75X coverage; mean read length = 15.68 Kb). Parent-specific k-mers (k=19), or “hap-mers”, were identified from whole-genome sequence data for ‘Cleopatra’ mandarin (*C. reticulata*) and ‘Flying Dragon’ (*P. trifoliata*) and used to phase the *Citrus* and *Poncirus* haplotypes in ‘US-897’ (Wu et al., 2018; Peng et al., 2020; Rhie et al., 2020; Rautiainen et al., 2023). For both haplotypes, the nine largest scaffolds had BUSCO scores of 99.0%, and captured 99.2% and 99.8% of parental hap-mers, an indication of assembly completeness (**Supplementary Table S1**). There was no evidence of haplotype switching within scaffolds, confirming that each haplotype was correctly phased in the diploid assembly of ‘US-897’ (**Figure 1A**). Haplotype 1 scaffolds were inherited from ‘Flying Dragon’ (*P. trifoliata*) (282.4 Mb, N50 = 29.9 Mb) and haplotype 2 scaffolds from ‘Cleopatra’ (*C. reticulata*) (321.4 Mb, N50 = 34.1 Mb) (**Figure 1A**; **Supplementary Table S1**). A similar number of genes were annotated in each haplotype (Haplotype 1: 33,431; Haplotype 2: 31,602 genes) and, based on BUSCO scores, the annotations included a majority of single copy orthologous genes used to benchmark genome annotations (Haplotype 1: 98.8%; Haplotype 2: 98.4%) (**Supplementary Table S1**). Orthology between the *Citrus* and *Poncirus* gene models was inferred based on protein sequence identity and collinearity using GeneTribe (Chen et al., 2020). This identified 22,419 reciprocal-best hits that were used for comparisons of allele-specific expression, along with 13,439 orthologous genes (single-best hits) and 6,756 singletons unique to one of the two parental haplotypes (**Figure 1B**). Analysis of allele-specific expression requires identification of gene orthology and this set of 22,419 genes were used to dissect the regulatory architecture of gene expression divergence between *Citrus* and *Poncirus*.

### Expression level dominance is pervasive in ‘US-897’

To better understand tolerance to HLB disease in ‘US-897’, we profiled the early transcriptional response of ‘Cleopatra’, ‘Flying Dragon’, and ‘US-897’ to *C*Las infection. The three genotypes were each infested by Asian citrus psyllids reared either on *C*Las-infected or clean citron plants (*C. medica*). Infection with CLas was confirmed for five ‘Cleopatra’, two ‘Flying Dragon’, and five ‘US-897’ individuals (**Supplementary Table S2**). To capture an early transcriptional response to CLas infection, leaf samples were collected from newly emerged branches 18 days after bud initiation. Previous work has shown early cellular and biochemical responses to CLas infection, including reactive oxygen species (ROS) production and callose deposition, occur as early as 15 days after inoculation (Ma et al., 2022). It is important to note that this sampling scheme captures initial host responses and the relationship between initial response and the robust tolerance of ‘US-897’ to Huanglongbing disease is unknown. An average of 28.3 million paired-end RNA-sequencing reads were generated per sample (**Supplementary Table S3**). RNA-seq reads were aligned to the haplotype-resolved genome of ‘US-897’ and the level of expression of the *Citrus* and *Poncirus* alleles of each gene were quantified using Salmon (Patro et al., 2017). In addition to allele-specific expression, levels of overall gene expression were determined by summing read counts for the *Citrus* and *Poncirus* alleles of each orthologous gene. Genes with low levels of expression were removed (CPM less than 2) and the remaining set of 20,981 genes were the basis of differential gene expression analysis.

**Figure 2:**
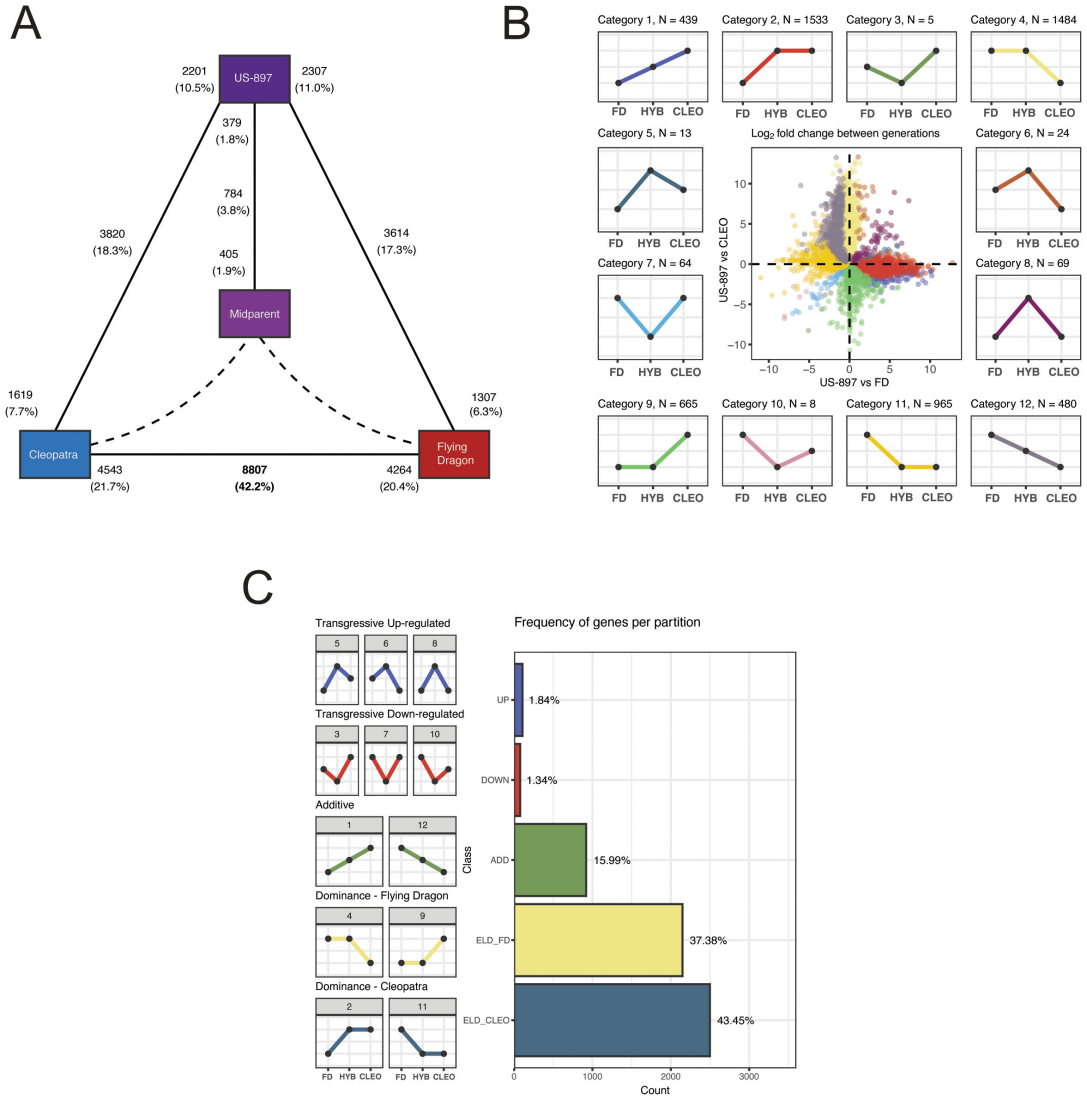
Classification of patterns of gene expression inheritance in ‘Cleopatra’, ‘US-897’, and ‘Flying Dragon’. **(A)** Diagram of the number of differentially expressed genes (DEGs) with a significant genotype effect in comparisons of: ‘Cleopatra’ versus ‘Flying Dragon’, ‘Cleopatra’ or ‘Flying Dragon’ versus ‘US-897’, and ‘US-897’ versus estimated mid-parent value. Solid black lines indicate comparison, with the total number of DEGs per comparison noted in the center of each line and the number of upregulated DEGs per comparison noted adjacent to each genotype. Percentages are based on the total number of tested genes (n = 20,981). **(B)** Categorization of patterns of gene expression inheritance. The focal dot-plot compares the log_2_ fold-change in gene expression between ‘US-897’ and its parents, with points colored by the subcategory of expression inheritance. The expression pattern of each category is shown in the 12 inset plots. **(C)** The 12 subcategories of gene expression inheritance were grouped into five major classifications: transgressive up in ‘US-897’, transgressive down in ‘US-897’, additive, dominant for ‘Flying Dragon’ allele, and dominant for ‘Cleopatra’ allele.

The analysis of gene expression in parent-offspring trios is essential for determining patterns of gene expression inheritance. Whether a gene exhibits additive, dominant, or transgressive patterns of inheritance in an F_1_ hybrid compared to its two parents can provide mechanistic insights into gene expression divergence between parental alleles. A linear model was constructed to explain overall gene expression by a genotype effect (‘Cleopatra’, ‘Flying Dragon’, ‘US-897’, and mid-parent value), a treatment effect (*C*Las-inoculated, mock-inoculated), and the interaction between genotype and treatment. A total of 9,522 differentially expressed genes (DEGs) with a significant genotype effect were identified (‘Cleopatra’ versus ‘Flying Dragon’, ‘Cleopatra’ or ‘Flying Dragon’ versus ‘US-897’, and ‘US-897’ versus estimated mid-parent value) (**Figure 2A**, FDR adjusted p-value < 0.05). A majority of these genes (8,807) were differentially expressed between the parental genotypes (**Figure 2A**). For genes with additive expression the level of expression in the hybrid is similar to the average of parental allelic expression, or the mid-parent expression value. Comparison of gene expression between ‘US-897’ and the mid-parent value revealed that gene expression patterns in the hybrid deviated from additivity for a set of 784 genes (**Figure 2A**).

The relative abundance of gene expression across all three genotypes was used to further categorize DEGs with significant genotype effects based on inheritance patterns (Rapp et al., 2009; Almeida-Silva et al., 2023). Genes with additive gene expression in the hybrid will be intermediate between the parental levels of expression (similar to the mid-parent value). Genes with non-additive gene expression in the hybrid will be biased towards expression of one of the two parental alleles (dominant) or is significantly higher or lower than expression of both parental alleles (transgressive). Gene expression patterns in each of these three categories can be further partitioned based on which parental allele is expressed at a higher level. DEGs were grouped into five categories (1 additive, 2 dominant, 2 transgressive) and 12 subcategories based on relative expression differences between the parental alleles (**Figures 2B, C**). For example, subcategories 1 and 12 both include genes with additive inheritance, but category 1 contains genes with reduced expression of the ‘Flying Dragon’ allele and category 12 includes genes with increased expression of the ‘Flying Dragon’ allele (**Figure 2C**). The majority of DEGs (80.83%) display expression level dominance, indicating that one of the two parental alleles is commonly favored in the hybrid ‘US-897’ (**Figure 2C**). Dominance of the maternal ‘Cleopatra’ allele was more common (43.45% of DEGs) than dominance of the paternal ‘Flying-Dragon’ allele (37.38% of DEGs) (**Figure 2C**). In *Arabidopsis thaliana* hybrids, expression level dominance in F_1_ hybrids was also common, affecting 90% of DEGs (Yuan et al., 2023). The preference for maternal expression bias has also been observed in other studies of parent-offspring trios hybrids (Videvall et al., 2016). Examples of DEGs with transgressive patterns of expression were rare (3.18%). Comparison of gene expression patterns in this parent-offspring trio revealed genes with additive, dominant, and transgressive expression patterns, but that expression level dominance was the most pervasive pattern of gene expression in ‘US-897’.

### Genes with additive and dominant patterns of gene expression are regulated in cis

**Figure 3:**
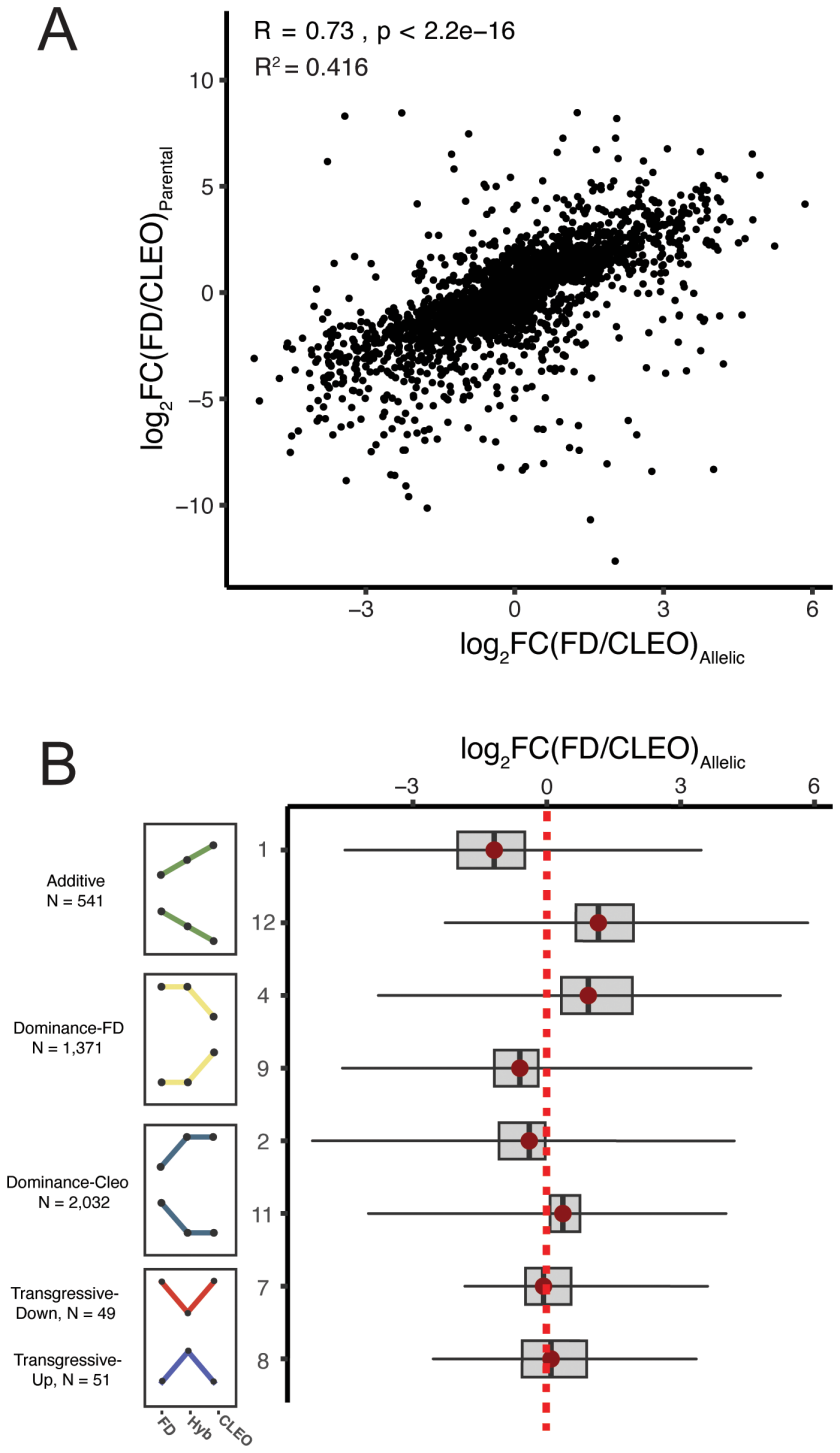
The role of cis-regulatory variation in expression divergence between *Citrus reticulata* and *Poncirus trifoliata*. **(A)** The relationship between ASE (log_2_(FD/CLEO)_Allelic_) and expression divergence between parental genotypes ‘Flying Dragon’ and ‘Cleopatra’ (log_2_(FD/CLEO)_Parental_). **(B)** The magnitude of ASE for genes with different patterns of gene expression inheritance. Categories 3, 5, 6, and 10 are not shown because there were no or few genes with ASE.

To better understand the evolution of gene regulation it is crucial to determine whether gene expression is controlled by cis-acting or trans-acting variants. Classification of cis- and trans-regulatory effects on gene expression can be determined by comparing the expression of parental alleles in an F_1_ hybrid. If differential expression between parental species (i.e. *C. reticulata* and *P. trifoliata*) is caused by a cis-regulatory variant, allele-specific expression of this gene is expected in the F_1_ hybrid. In contrast, a trans-regulatory variant would cause differential gene expression between parental species, but concordant expression of both parental alleles in the hybrid. Cis-regulatory variants are generally considered to be major contributors to expression divergence between different species, while trans-regulatory variants are more commonly associated with expression differences within species (Emerson et al., 2010; Metzger et al., 2017; Signor and Nuzhdin, 2018; Zhang and Emerson, 2019; Coolon et al., 2014). To determine if gene expression is controlled in cis or trans we compared DEGs with significant genotype effects to allele-specific expression (ASE) of parental alleles in ‘US-897’. Genes with ASE in ‘US-897’ were identified using the same set of 22,419 orthologous genes between *Citrus* and *Poncirus* haplotypes. After removing genes with low expression, monoallelic expression, and insufficient genetic variation (a limitation in the analysis of ASE), ASE could be evaluated for 15,549 genes. Allele-specific expression was common in ‘US-897’ (9,189 genes, FDR-corrected p < 0.05), indicating that cis-regulatory variation has a major role in regulating hybrid gene expression patterns. On average, there was a 97% difference in expression between parental alleles for genes with significant ASE (log_2_(Haplotype 1 (*P. trifoliata*)/Haplotype 2 (*C. reticulata*)) = 0.98). Notably, allele-specific expression in ‘US-897’ explains 41.6% of differential gene expression variation between ‘Cleopatra’ and ‘Flying Dragon’ (**Figure 3A**; Spearman’s rho = 0.74, R^2^ = 0.416). Parental haplotypes have diverged for ~9 million years (Wu et al., 2018; Huang et al., 2023b), and our results are consistent with the role of cis-regulatory variation in divergence of gene expression patterns.

Next, we explored if cis-regulated genes (i.e. ASE genes in ‘US-897’) corresponded to additive, dominant, or transgressive patterns of expression inheritance. On average, the allele expressed more highly in the hybrid was consistent with parental expression patterns. For example, additively expressed genes with higher expression in ‘Cleopatra’ (Category 1) exhibit allele expression bias towards the ‘Cleopatra’ allele in ‘US-897’ (**Figure 3B**). This trend is consistent for all patterns of additive and dominant gene expression categories (**Figure 3B**). Allele-specific expression was not detected for genes with transgressive expression in the F_1_ hybrid consistent with trans-regulation of this gene set (**Figure 3B**). Additionally, genes with the greatest magnitude of allele-specific expression are additively inherited, perhaps with cis-regulatory variants of large effect. The abundance of genes with additive and dominant patterns of expression inheritance and their allelic dynamics in ‘US-897’ is consistent with the major contribution of cis-regulatory variation to gene expression divergence between *Citrus* and *Poncirus*.

### Genes responsive to *C*Las infection have reduced signature of cis-regulation

**Figure 4:**
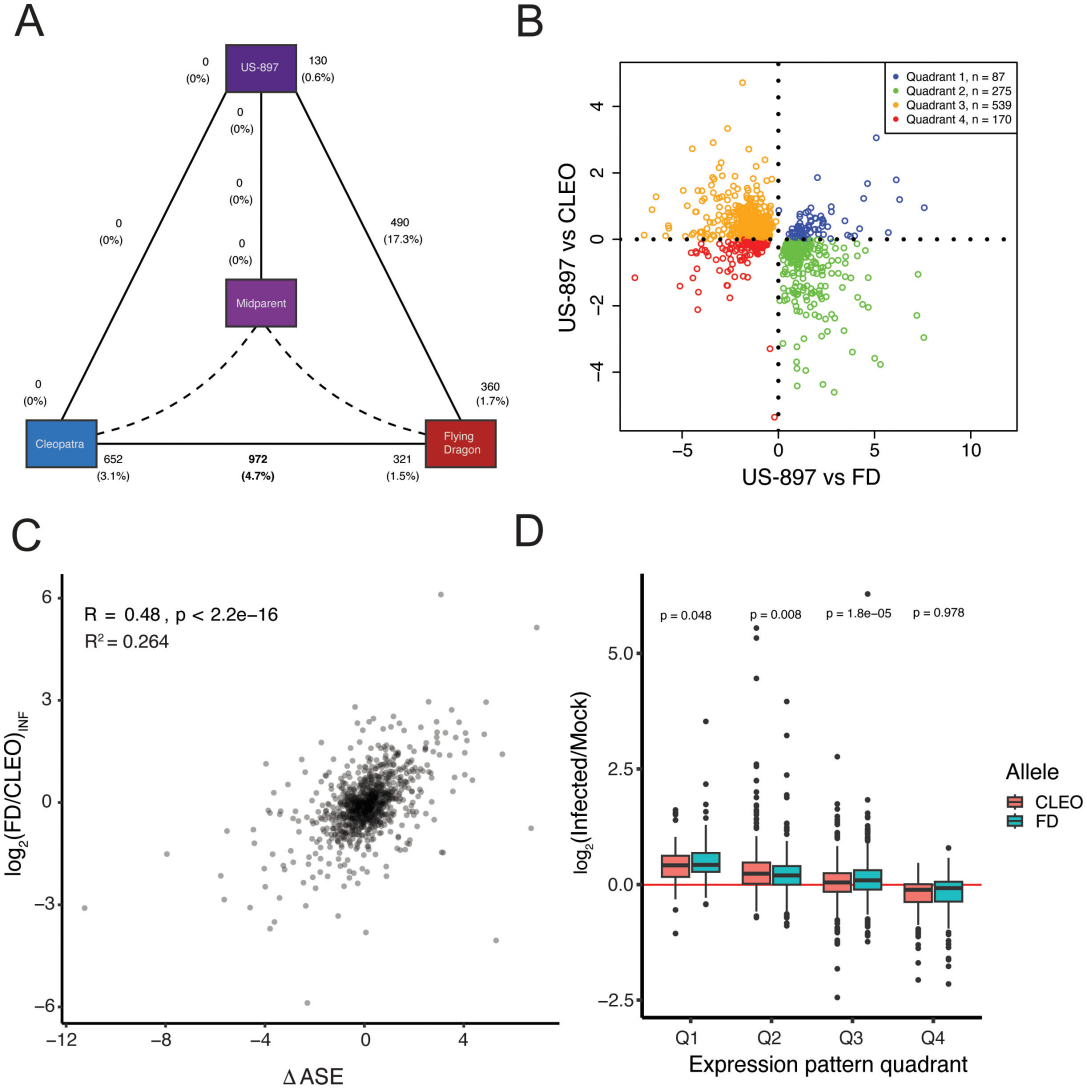
Classification of patterns of gene expression inheritance in ‘Cleopatra’, ‘US-897’, and ‘Flying Dragon’ for genes with significant genotype x treatment interactions. **(A)** Diagram of the number of differentially expressed genes (DEGs) in comparisons of: ‘Cleopatra’ versus ‘Flying Dragon’, ‘Cleopatra’ or ‘Flying Dragon’ versus ‘US-897’, and ‘US-897’ versus estimated mid-parent value. Solid black lines indicate comparisons, with the total number of DEGs noted in the center of each line and the number of upregulated DEGs noted adjacent to each genotype. Percentages are based on the total number of test genes (n = 20,981). **(B)** Categorization of gene expression patterns relative to expression in ‘US-897’ versus parental genotypes for genes with significant genotype x treatment interaction (n=1,071). The dot-plot compares the log_2_ fold-change in gene expression between infected samples of ‘US-897’ and its two parents, with points colored by quadrant. **(C)** The relationship between Δ ASE in ‘US-897’ (the allelic response to *C*Las infection) (log_2_(FD/CLEO)_Allelic_) and expression divergence between parental genotypes ‘Flying Dragon’ and ‘Cleopatra’ (log_2_(FD/CLEO)_Parental_). Only genes with significant genotype x treatment effects that could be tested for ASE are included (n=975). **(D)** The response of each allele in ‘US-897’ to pathogen infection for genes in each of the quadrants in 4B. Only genes with significant genotype x treatment effects that could be tested for ASE are included (n=975).

The relative importance of cis- and trans-regulation to environmental response is still unclear. There is evidence of both cis- and trans-acting regulation of gene expression in response to environmental signals (Snoek et al., 2017; Reynoso et al., 2019; Zeng et al., 2019; Zhou et al., 2021), although some suspect that trans-regulators may be more critical for environmental response (Signor and Nuzhdin, 2018). To gain insight into the role of cis- versus trans-regulation of genes conferring HLB-tolerance in ‘US-897’, we first identified genes with significant treatment effects and genotype x treatment interactions. Only 629 genes were identified with a significant treatment effect (FDR corrected p < 0.05, **Supplementary Table S4**). This set of genes is responsive to *C*Las infection regardless of genotype. Of these 629 genes, 245 were upregulated, and 384 were downregulated (**Supplementary Figure S1A**). The few genes responding to *C*Las infection here may be due to the early sampling only 18 days after bud initiation. Previous gene expression analysis comparing ‘Cleopatra’ to ‘US-897’ identified many more differentially expressed genes, but plants were sampled 7 to 8 months after infection (Albrecht and Bowman, 2012b). While early transcriptomic changes are important for understanding the initial stages of host-pathogen interactions, they do not explain the full suite of mechanisms underlying tolerance to Huanglongbing disease. Gene ontology enrichment analysis revealed that up-regulated genes were enriched for several biological processes related to regulation of the cell cycle, while down-regulated genes were enriched for processes related to cutin biosynthesis, cell wall organization, and lipid metabolism (**Supplementary Table S5**; Fisher’s exact test, p < 0.001). A larger group of genes with a significant genotype x treatment interaction were identified. Of the 1,071 genes with significant genotype-specific responses to HLB disease (FDR corrected p < 0.05), a majority (972) exhibited differential response to *C*Las infection between the two parental genotypes, ‘Cleopatra’ and ‘Flying-Dragon’. A smaller number of genes (490) were differentially responsive to *C*Las between one parent, ‘Flying-Dragon’, and ‘US-897’ (**Figure 4A**; FDR corrected p < 0.05). There was some overlap between genes with significant genotype x treatment interactions between contrasts. Surprisingly, no genes were differentially responsive to *C*Las between the second parent, ‘Cleopatra’ and ‘US-897’. The two parents, ‘Flying Dragon’ and ‘Cleopatra’, are responding differently to infection with *C*Las, at least in the early stages of infection. Few genes are differentially responsive to *C*Las in the hybrid compared to its two parents, suggesting that much of the parental response to infection was inherited in the hybrid.

To assess the contribution of cis-regulation to *C*Las responsive genes we quantified the response in allele-specific expression in ‘US-897’ (ΔASE) compared to expression divergence between the infected parents. Of the 1,071 genes with significant genotype x treatment effects, 975 could be assessed for allele-specific expression in ‘US-897’. For these genes there is a moderate correlation between ΔASE and differential expression between infected parents (**Figure 4C**, Spearman’s rho = 0.48, R^2^ = 0.264) with Δ ASE explaining 26.4% of variation in divergent expression between ‘Flying Dragon’ and ‘Cleopatra’. We also examined the relationship between ΔASE and differential expression between infected parents for 536 of the 629 genes with significant treatment effects. For these genes, ΔASE explained an even smaller proportion of variation in divergent expression between parents (R^2^ = 0.134; **Supplementary Figure S1C**). This suggests that cis-regulatory variants are also a component of divergence in disease response, but that the signature of cis-regulation is reduced.

To further investigate cis-regulation of genes responding to *C*Las infection, the allelic response to pathogen infection was evaluated in ‘US-897’. Genes were partitioned into quadrants that were based on whether they were up- or down-regulated between ‘US-897’ and its parents (**Figure 4B**). Allele-specific responses to pathogen infection were observed for three of the four quadrants for genes with a significant genotype x treatment interaction (**Figure 4D**; paired t-test, p < 0.01) and two of four quadrants for genes with significant treatment effect (**Supplementary Figure S1D**; paired t-test, p < 0.01). Overall, the signature of cis-regulation of pathogen-responsive genes is reduced compared to genes with significant genotype effects (**Figure 3B**), but cis-regulation still contributes to the transcriptional response to *C*Las infection.

### Cis-regulation of genes involved in plant immunity

**Figure 5:**
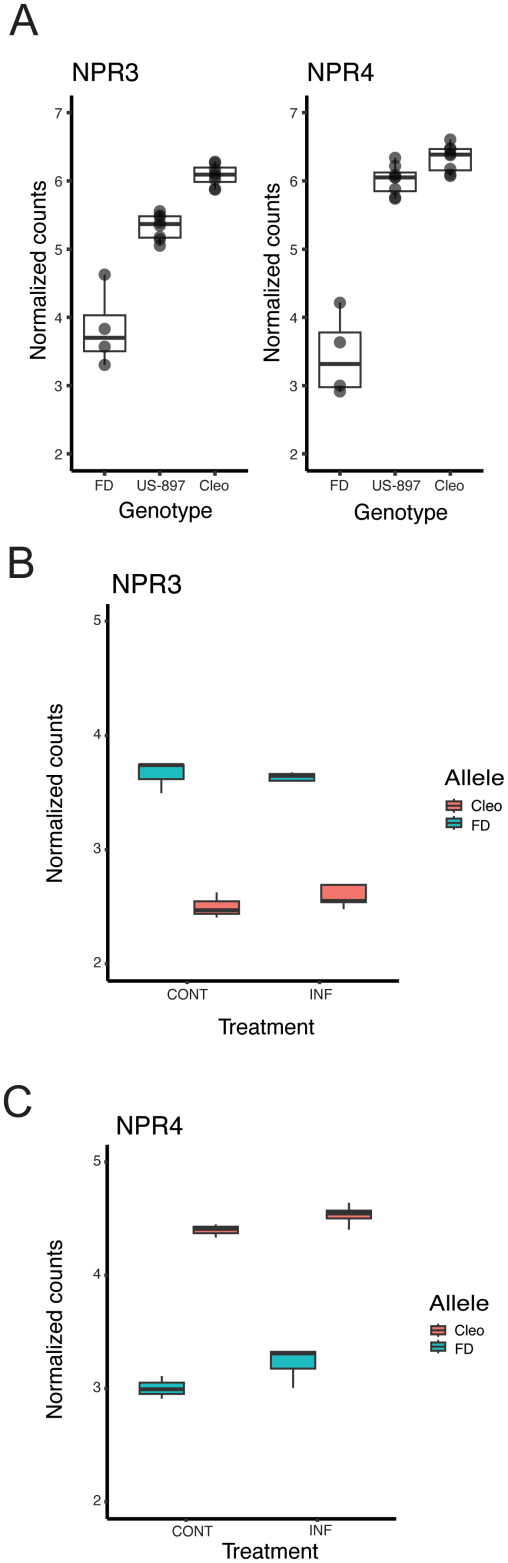
Cis-regulation of NPR3 and NPR4 expression in ‘US-897’. **(A)** The expression of homologs of NPR3 (left) and NPR4 (right) in ‘Flying Dragon’ (n=4), ‘US-897’ (n=8), and ‘Cleopatra’ (n=8). These genes are significantly differentially expressed in ‘US-897’ compared to both of its parents (FDR adjusted p-value < 0.05). **(B)** Allele-specific expression of NPR3 in ‘US-897’ in mock and infected treatments. **(C)** Allele-specific expression of NPR4 in ‘US-897’ in mock and infected treatments.

Genes with the largest magnitude of allele-specific expression in ‘US-897’ were additively inherited, possibly due to large-effect cis-regulatory variants. Notably, genes with additive inheritance favoring expression of the ‘Cleopatra’ allele were significantly enriched for genes involved in systemic acquired resistance (SAR) (two out of seven annotated genes; p < 0.01; **Supplementary Table S6**). These two genes are homologs of NONEXPRESSOR OF PATHOGENESIS-RELATED3 (AtNPR3) and NONEXPRESSOR OF PATHOGENESIS-RELATED4 (AtNPR4) in *Arabidopsis thaliana* and are negative regulators of immunity and critical components of plant defense against pathogens (Ding et al., 2018; Zhang et al., 2006). NPR3 has also been shown to negatively regulate immunity in sweet orange, where silencing of NPR3 expression increased basal callose levels and reduced pathogen-induced callose deposition and ROS accumulation (Sarkar et al., 2024). The expression of both NPR3 and NPR4 in ‘US-897’ was intermediate between ‘Cleopatra’ and ‘Flying Dragon’ (**Figure 5A**). We reasoned that reduced expression of NPR3 and NPR4 in ‘US-897’ could enhance basal immunity in the hybrid compared to its susceptible parent ‘Cleopatra’. To understand how novel combinations of cis-regulatory alleles affect expression of these genes, we evaluated allele-specific expression in ‘US-897’. Surprisingly, intermediate expression of NPR3 in ‘US-897’ results from high expression of the ‘Flying Dragon’ allele and low expression of the maternal ‘Cleopatra’ allele (**Figure 5B**, FDR corrected p < 0.01). This contrasts with the parental expression patterns, where ‘Cleopatra’ expression is high and ‘Flying Dragon’ expression is low (**Figure 5A**). The patterns of allelic expression differ at NPR4 with low expression of the ‘Flying Dragon’ allele and high expression of the ‘Cleopatra’ allele resulting in intermediate expression of the gene in ‘US-897’ compared to its two parents (**Figure 5C**). Patterns of allelic expression of NPR3 and NPR4 in ‘US-897’ are consistent for uninfected and *C*Las infected treatments. NPR3 and NPR4 negatively regulate NPR1, but because this regulation occurs post-transcriptionally (Fu et al., 2012), no relationship between levels of gene expression between NPR1 and NPR3/4 are expected. We did find that ‘Flying Dragon’ and ‘US-897’ have significantly lower NPR1 expression than ‘Cleopatra’ (**Supplementary Figure S3**). Overall, expression of NPR3 and NPR4 is reduced in ‘US-897’ compared to its susceptible parent, ‘Cleopatra’, and both genes have signatures of cis-regulation.

## Discussion

Parent-offspring trios are commonly used to infer patterns of inheritance and, in the case of gene expression, are essential for dissecting cis- and trans- gene regulatory architecture (Hill et al., 2021). We selected a parent-offspring trio with relevance to a current major disease impacting worldwide production of citrus (Albrecht and Bowman, 2011; Court and Rahmani, 2017). The interspecific hybrid, ‘US-897’ is a commercial cultivar with tolerance to HLB disease (Albrecht and Bowman, 2011). Analysis of differential gene expression between parents and hybrid and allele-specific expression in the hybrid was made possible by the production of a phased, diploid assembly of ‘US-897’. The combination of PacBio HiFi sequencing with kmer-based trio-binning resolved both parental haplotypes of the ‘US-897’ genome, resulting in the first published reference genome containing both *Citrus* and *Poncirus* ancestry.

### Genes with additive and dominant expression are cis-regulated

Expression level dominance was the most prevalent pattern of gene expression in ‘US-897’ compared to its parents. More than 80% of genes with significant differential expression between genotypes, in both untreated (**Figure 2C**) and treated samples (**Supplementary Figure S2**), were dominantly expressed in the hybrid. This is consistent with other parent-offspring trios, including in *Arabidopsis thaliana* F_1_ hybrids, where expression dominance was also detected at the majority of genes differentially expressed between hybrid and parents (Yuan et al., 2023). Interestingly, in untreated samples, dominant gene expression was slightly biased towards expression of the maternal allele (‘Cleopatra’) (**Figure 2C**), but this trend reversed in *C*Las treated samples, instead favoring dominant expression of the paternal allele (‘Flying Dragon’) (**Supplementary Figure S2**).

Allele-specific expression of parental alleles in hybrid genomes is an indicator of cis-regulatory control of gene expression. Analysis of allele-specific expression in ‘US-897’ revealed that a majority of genes differentially regulated between ‘Cleopatra’ and ‘Flying Dragon’ are regulated in cis. In addition to the large number of genes expressed in ‘US-897’ with ASE (59%), there was a strong correlation between ASE in ‘US-897’ and differential expression between the two parents (**Figure 3A**). Together this suggests that divergence in gene expression between the *Citrus reticulata* (‘Cleopatra’) and *Poncirus trifoliata* (‘Flying Dragon’) primarily results from cis-regulatory variation. Genes with the greatest magnitude of ASE in ‘US-897’ were those genes with additive inheritance and dominant inheritance with higher expression of the ‘Flying Dragon’ allele (**Figure 3B**). Strong allele-specific expression of these genes could be caused by large-effect mutations in cis-regulatory regions, and mining the regulatory regions of these genes could reveal regulatory motifs that have diverged between *Citrus* and *Poncirus*.

We focused on additively inherited genes with strong allele specific expression and discovered significant enrichment for genes involved in systemic acquired resistance (SAR), including homologs of *Arabidopsis thaliana* NONEXPRESSOR OF PATHOGENESIS-RELATED3 (AtNPR3) and NONEXPRESSOR OF PATHOGENESIS-RELATED3 (AtNPR4). These genes have established roles as negative regulators of plant immune responses (Ding et al., 2018; Zhang et al., 2006), including recently in citrus where silencing of NPR3 increased tolerance to HLB in sweet orange, suggesting that modulating NPR3 activity could tune the immune response to mitigate disease (Sarkar et al., 2024). Reduced expression of NPR3 and NPR4 in ‘US-897’ compared to susceptible ‘Cleopatra’ suggests that the hybrid may contain a favorable immune status compared to its susceptible parent. Further dissecting allele-specific expression (ASE) of NPR3 and NPR4 in ‘US-897’ revealed complex underlying regulatory architectures. For NPR4, we found evidence of a classic example of cis-regulation, with low-expression of the ‘Flying Dragon’ allele and higher expression of the ‘Cleopatra’ allele resulting in intermediate expression of NPR4 in the hybrid. In contrast, the regulatory control of NPR3 was more complex with intermediate expression of NPR3 in ‘US-897’ resulting from unexpectedly high expression of the ‘Flying Dragon’ and low expression of the ‘Cleopatra’ allele. This suggests an interplay of cis- and trans-acting factors, but the expression of each allele would need to be evaluated in both parental backgrounds to further dissect the regulation of NPR3. The precise implications and physiological impact of cis-regulation of NPR3 and NPR4 and their effect on HLB tolerance will require further functional evaluation.

### Transgressive gene expression was limited in ‘US-897’

Genes with transgressive expression should not exhibit ASE, because trans-regulators will affect the expression of both parental alleles in the hybrid. Indeed, genes with transgressive expression in ‘US-897’ were not expressed in an allele-specific manner (**Figure 3B**). Overall, very few genes were transgressively expressed in both untreated and *C*Las-treated samples (**Figure 2C**; **Supplementary Figure S2**). Transgressive expression could be a consequence of hemizygosity, with the hybrid inheriting two or zero copies of a gene and resulting in transgressive up or downregulation in ‘US-897’, respectively. In fact, 10 - 15% of genes are hemizygous in the clonally propagated grapevine (*Vitis* vinifera) and cassava (*Manihot esculenta*) (Zhou et al., 2019; Peng et al., 2025). Similarly, in ‘US-897’, more than 6,000 genes were present in only one of the two parental haplotypes, making this a promising avenue for future research. Genes with transgressive expression have long been of interest for their potential contribution to hybrid vigor, but overall we find that transgressive expression is limited in this interspecific hybrid.

### Genes responsive to *C*Las infection have a reduced signature of cis-regulation

Cis-regulatory sequences are also important for regulating gene expression in response to environmental signals (Reynoso et al., 2019; Zeng et al., 2019; Zhou et al., 2021). For example, in two species of *Arabidopsis* more than 6,000 drought responsive cis-regulatory variants were identified (He et al., 2021). To explore the contribution of cis-regulation to the response to *C*Las infection, genes with a significant treatment effect (n=629) or genotype x treatment interaction (n=1,071) were identified. Compared to previous studies of gene expression in ‘US-897’ and its maternal parent 7–8 months after infection (Albrecht and Bowman, 2012b), relatively few *C*Las responsive genes were identified at just 18 days after bud initiation.

Cis-regulation of genes responsive to *CLas* infection was limited compared to cis-regulation of gene expression divergence between ‘Cleopatra’ and ‘Flying Dragon’. This is supported by the reduced correlation of ΔASE in ‘US-897’ (the allelic response to *C*Las infection) to parental responses to *C*Las infection (**Figure 4C**; **Supplementary Figure S1C**). Also, evaluation of allelic response to *C*Las revealed that there were significant differences between the response of the maternal or paternal allele in ‘US-897’, although the differences were subtle (**Figure 4D**). This suggests that cis-regulatory variants are important for the transcriptional response to pathogen infection, but their contribution may be reduced for pathogen-responsive genes.

### Relevance of genetic architecture for breeding new citrus cultivars

Many citrus rootstocks in current use for citriculture throughout the world are hybrids of *Citrus* species with *Poncirus trifoliata* (Bowman and Joubert, 2020). *Poncirus trifoliata* has been highly valued as a parent to create new rootstocks because of several important traits that are transmitted to progeny, including nucellar polyembryony (Kepiro and Roose, 2009), tolerance of citrus nematodes (Ling et al., 2000; Imran Hamid et al., 2025), tolerance of *Phytophthora* root rot (Graham, 1995), resistance to citrus tristeza virus (Fang et al., 1998), and cold tolerance (Yelenosky, 2011). More recently, *P. trifoliata* has also been recognized as transmitting *C*Las-resistance and tolerance to progeny that are useful as rootstocks (Albrecht and Bowman, 2011, 2012b), and breeding work has expanded to include *P. trifoliata* ancestry in advanced-generation rootstock hybrids with tolerance to *C*Las (Bowman et al., 2021; Bowman, 2023).

Although less widely recognized, *P. trifoliata* has also been incorporated in citrus scion breeding for decades, to introduce resistance to tristeza virus and better cold hardiness into new scions (Young et al., 1982; Tignor et al., 1998). More recently, the aim of such efforts has shifted to include breeding new scion hybrids that contain tolerance to *C*Las from *P. trifoliata* ancestry (Huang et al., 2023a). In citrus scion breeding, first generation hybrids with trifoliate orange typically have significant undesirable flavor components in fruit juice (Deterre et al., 2021). However, backcrossing of these *P. trifoliata* orange hybrids to citrus for one or more generations, combined with progeny selection, can eliminate these off-flavors, and recover new hybrids that combine the desired *C*Las-tolerance traits with good-flavored fruit (Fang et al., 1998; Jeffries et al., 2024). In breeding of both citrus rootstocks and citrus scions, information about regulation of gene expression for critical traits is severely limited, and lack of knowledge about transmission of key traits from parents to progeny greatly hampers progress in developing new cultivars with those essential traits. A clear understanding about genetic architecture and the regulation of gene expression associated with *C*Las infection will dramatically improve opportunities to make meaningful gains in the breeding of both rootstocks and scions with tolerance to *C*Las. While our transcriptomic analyses provide insights into the regulation of gene expression in response to *C*Las infection, the molecular basis of HLB tolerance is still unknown and will require validation of the roles of specific regulatory variants and genes in conferring HLB tolerance.

## Conclusion

Cis-regulatory variants are a common and significant contributor to expression divergence between *Citrus* and its wild relative *Poncirus*. These cis-acting variants are associated with additive and dominant gene expression inheritance. But, genes responding to *C*Las shortly after infection have a reduced signature of cis-regulation, suggesting a dynamic interplay between cis- and trans-regulation of response to *C*Las infection in the interspecific hybrid, ‘US-897’. The high-quality, haplotype-phased genome assembly of ‘US-897’, alongside the insights gained from analysis of allele-specific expression in the ‘US-897’, provide a critical resource for uncovering the genetic basis of HLB tolerance in *Citrus* x *Poncirus* hybrids.

## Materials and methods

### Plant cultivation and *C*Las inoculation

*Candidatus liberibacter asiaticus* (*C*Las) is a quarantined pathogen in California and all plant cultivation and psyllid-mediated inoculations were performed in the California Citrus Research Foundation containment facility located in Riverside, CA. Seeds of ‘Cleopatra’ mandarin (*Citrus reticulata*), ‘Flying Dragon’ trifoliate orange (*Poncirus trifoliata*), and their F_1_ hybrid ‘US-897’ were sterilized prior to germination. The primary seed coats were removed, and seeds were washed with 70% isopropanol for 10 minutes. Next, seeds were treated with a 10% bleach solution for 30 minutes and rinsed three times with sterile water. Seeds were placed in 15 ml culture tubes containing seed germination media (0.22% Murashige and Skoog basal salt mixture (Murashige and Skoog, 1962), 2.5% sucrose, 0.005% myo-inositol, 0.50% ethylenediaminetetraacetic acid ferric salt, and 0.70% agar) and grown at 30° C in the dark for two weeks. After germination, seeds were transplanted to cone-tainers (164 ml) with coconut coir and placed in a greenhouse with 12 hour supplemental light:dark cycles with light intensity maintained at ~ 500 µmol/m²/s. Both ‘Cleopatra’ and ‘Flying Dragon’ produce nucellar seeds. All transplanted seedlings were genotyped with 24 LGC KASP™ markers (Hoddesdon, UK) and zygotic seedlings were culled.

For *C*Las inoculation, Asian citrus psyllids (*Diaphorina citri*) were reared on either confirmed CLas-infected citron (*Citrus medica*) plants or CLas-free citron plants, thereby providing CLas-positive (CLas+) and mock-inoculation (CLas-) psyllid populations, respectively. Nine month old plants (6 replicates) of each genotype (‘Cleopatra’, ‘Flying Dragon’, US-897) were infested with 15–20 CLas+ psyllids per plant for a period of four weeks. Concurrently, three replicate plants of each genotype were similarly infested with 15–20 clean (CLas-) psyllids per plant to serve as mock-inoculated controls. Psyllids were contained on the plants using small mesh cages enclosing a single branch during the infestation period. In some rare cases, smaller plants were completely caged. Plants were nine months-old at the time of infestation with psyllids. Before tissue sampling for RNA-sequencing, all experimental plants were pruned to stimulate new flush. At this time, the systemic *C*Las infection status of each individual plant was confirmed by quantitative PCR of the *nrdB* gene of *C*Las as performed in (Zheng et al., 2016). The leaf sampled to confirm CLas infection was from the single, caged branch (or small plant). This branch was trimmed and then bud initiation was tracked, with sampling of leaf tissue for RNA-seq occurring 18 days after bud initiation. Based on qPCR results, five ‘Cleopatra’, two ‘Flying Dragon’, and five US-897 individuals from the CLas+ treatment group were confirmed as infected and used for subsequent RNA-seq analysis (**Supplementary Table S2**). All plants, mock- and CLas-infected, were randomized in both the plant growth chamber, where psyllid inoculations occurred, and in the greenhouse.

### Sampling scheme and RNA-sequencing

Plants were transferred from the growth chamber used for psyllid-mediated *C*Las (and mock) inoculations to the greenhouse two months after their initial exposure to psyllids. Plants continued to grow for an additional two months in the greenhouse and then, after adjusting to greenhouse conditions, leaf samples were collected to assay for *C*Las. This enables sufficient establishment of the bacteria in the host plants. Additionally, *C*Las detection in new shoots has been shown to occur as early as 15 days post-bud initiation (Ma et al., 2022). After sampling, a single branch on each plant was trimmed to induce new flush and plants were monitored daily for bud initiation. Next, leaf tissue from the newly emerging flush was sampled 18 days post bud initiation to capture early responses to pathogen infection as performed in (Ma et al., 2022). While the exact newly emerging shoots sampled for RNA-seq were not individually quantified for bacterial titer, CLas is known to be transported to and can be detected in newly developing flush tissues within 15 days of bud-initiation (Ma et al., 2022). Therefore, RNA-seq was performed on these newly emerged shoots from systemically infected plants. All sampling was performed from 10-11AM Pacific Standard Time (PST). After sampling, leaf tissue was flash frozen in liquid nitrogen and quarantined at -80° C for 14 days. RNA extraction was then performed using the Qiagen RNeasy Plant kit (Hilden, Germany). Sequencing libraries were prepared using the Illumina Stranded mRNA prep, Ligation kit (Pleasanton, California). Libraries were sequenced on two lanes of a NovaSeq X Plus (PE150) by Novogene (Beijing, China) generating an average of 28.3 million reads per sample (**Supplementary Tables S3**, **S4**).

### PacBio sequencing

Nuclei extraction from ~3 grams of young leaf tissue from a single individual of the F_1_ hybrid ‘US-897’ was performed using PacBio’s nuclei extraction protocol (PacBio, 2022b). High molecular weight DNA (HMW DNA) was then extracted from nuclei using PacBio’s Nanobind plant kit (PacBio, 2022a). An extended lysis step was included, increasing from 30 minutes to 2 hours. A second lysis step was performed to ensure sufficient extraction of HMW DNA. HMW DNA was then sent to Novogene (Beijing, China) for library preparation and sequencing on one SMRT cell of the PacBio Revio (Menlo Park, California). This generated 65 Gb of HiFi sequencing reads, with an average read length of 15.6 Kb.

### Genome-assembly and annotation

PacBio HiFi sequencing reads were used to assemble the diploid genome of the F_1_ hybrid ‘US-897’. First, Illumina whole-genome sequencing reads from the parental genotypes, ‘Cleopatra’ mandarin and ‘Flying Dragon’ trifoliate orange (Wu et al., 2018; Peng et al., 2020), were used to identify parent-specific k-mers (k=19), or hap-mers, using Merqury (Rhie et al., 2020). PacBio HiFi reads were error-corrected and used to construct an assembly graph using verkko (Rautiainen et al., 2023). Next, paths in this assembly graph were haplotype-resolved using parental hap-mer profiles (verkko trio), and a consensus algorithm was applied to construct the final diploid assembly (Rautiainen et al., 2023). Before genome annotation, repetitive sequences were soft-masked using a custom library of repeat sequences with RepeatMasker v 4.1.5 (Smit et al., 2013-2015). Genome annotation was then performed with BRAKER v3.0.3, using a custom database of proteins, including those specific to the genus *Citrus*. Only the 9 largest scaffolds of each haplotype were annotated. BUSCO scores were calculated for the genome assembly (-m geno) and the genome annotation (-m prot) using the eudicots_db10 database and Busco v5.8.0 (Manni et al., 2021).

### Differential gene expression analysis

Genome annotations for each haplotype of ‘US-897’ were used to construct a diploid transcriptome using Salmon v1.7.0 (Patro et al., 2017). Homology of transcripts between haplotypes was inferred based on protein sequence identity and collinearity using GeneTribe (Chen et al., 2020). Transcripts were classified based on whether they are reciprocal best hits (RBH) that are allelic between haplotypes, single best hits (SBH) are orthologous sequences, and singletons are genes that are unique to a haplotype. For ortholog identification, the gene IDs from the ‘Flying Dragon’ haplotype were used, unless the gene was only present in Cleopatra, then the gene IDs from the ‘Cleopatra’ haplotype were used. Next transcripts were quantified using Salmon v1.7.0 (Patro et al., 2017) with the following parameters: -validateMappings –numBootstraps 30 –recoverOrphans –writeUnmappedNames –allowDovetail –softclip –minScoreFraction 0.6. Bootstrapping (n=30) was performed to generate inferential replicates for accurate quantification of transcripts from RNA-sequencing reads. For analyses of overall gene-expression (as opposed to allele-specific expression), read counts for transcripts were summed for the set of orthologous genes. Gene expression estimates (Counts-Per-Million or CPM) were log_2_-transformed and weights were assigned to each observation based on the estimated mean-variance trend across genes using voom transformation (Law et al., 2014). Next, gene expression estimates for the estimated mid-parent values for each gene were calculated by averaging read counts of parental samples that were randomly selected from replicates of each treatment. This process was repeated to generate four replicates of mid-parent expression per gene. Genes with low levels of expression were removed (CPM less than 2) and the remaining set of 20,981 genes were the basis of differential gene expression analysis. Gene expression estimates were modeled using limma-voom (Law et al., 2014), including fixed effects for genotype (‘Cleopatra’, ‘Flying Dragon’, ‘US-897’, mid-parent), treatment (*C*Las-inoculated, mock-inoculated), and the genotype x treatment interaction. This mid-parent value-based classification, performed using limma’s linear models and statistical comparisons (Ritchie et al., 2015), serves as a standard approach for broadly categorizing gene expression inheritance patterns (Rapp et al., 2009; Huang et al., 2016). While the midparent value establishes a null hypothesis for additive inheritance for each gene, the use of limma accounts for intra-genotypic variability in its variance estimation, thereby providing robust statistical significance for deviations.

### Classification of hybrid gene expression inheritance patterns

Specific contrasts were evaluated to identify differentially expressed genes (DEGs) between genotypes (‘Cleopatra’ versus ‘Flying Dragon’, ‘Cleopatra’ or ‘Flying Dragon’ versus ‘US-897’, and ‘US-897’ versus estimated mid-parent value). Gene expression inheritance patterns were determined for genes with differential expression in any of these contrasts using the R package “HybridExpress” (Almeida-Silva et al., 2023). Significant differences based on Benjamini-Hochberg FDR < 0.05 and the log_2_(fold-change) for each contrast were used to classify gene-expression patterns. Genes were classified as having “additive” inheritance if there was no significant difference in expression (Benjamini-Hochberg FDR > 0.05) between the expected mid-parent value and ‘US-897’. Genes were classified as “dominant” if there was a significant difference between the hybrid and one parent, but not the other. Lastly, genes with significantly higher or lower gene expression in the ‘US-897’ compared to both parents were classified as transgressive. This same classification scheme was used to assess gene expression inheritance patterns for genotype x treatment contrasts.

### Identification of allele-specific expression in ‘US-897’

Gene expression was quantified as described above using Salmon, with bootstrapping (n=30) to generate inferential replicates for accurate quantification of alleles from RNA-sequencing reads. Gene expression was quantified for 22,419 genes, and genes with read counts greater than ten in at least six samples were retained (n=17,487). Genes for which inferential replicates of each allele were almost identical were filtered from downstream analyses (n=2), as this indicates there is no information about allelic expression in the sequencing reads. For each gene, the ratio of allele 1 (‘Flying Dragon’): allele 2 (‘Cleopatra’) expression was averaged across all samples, and genes with a ratio greater than 0.95 (n=371) or less than 0.05 (n=467) were classified as having monoallelic expression. Monoallelic genes were excluded from modeling of allele-specific expression resulting in 15,549 genes that could be evaluated for allele-specific expression.

## Data Availability

All raw sequencing data has been deposited at NCBI, including RNA-sequencing reads (BioSamples: SAMN50643246-SAMN50643264, SAMN506432650) and PacBio HIFI reads used to assemble the genome of 'US-897' (BioSample: SAMN50643266). The genome assembly of 'US-897' has also been deposited at NCBI under BioProject PRJNA1293460 and PRJNA1293461 for the paternal and maternal haplotypes, respectively.

## Supplementary material

Supplementary Figure 1 Classification of patterns of gene expression inheritance in ‘Cleopatra’, ‘US-897’, and ‘Flying Dragon’ for genes with significant treatment effects (n=629). **(A)** Identification of genes with significant treatment effects. The dotted line represents the significance threshold (FDR corrected p < 0.05). **(B)** Categorization of gene expression patterns relative to expression in ‘US-897’ versus parental genotypes for genes with significant genotype x treatment interaction (n=629). The dot-plot compares the log_2_ fold-change in gene expression between infected samples of ‘US-897’ and its two parents, with points colored by quadrant. **(C)** The relationship between Δ ASE in US-897 (the allelic response to *C*Las infection) (log_2_(FD/CLEO)_Allelic_) and expression divergence between parental genotypes ‘Flying Dragon’ and ‘Cleopatra’ (log_2_(FD/CLEO)_Parental_). Only genes with significant treatment effects that could be tested for ASE are included (n=535). **(D)** The response of each allele in ‘US-897’ to pathogen infection for genes in each of the quadrants in **(B)**. Only genes with significant treatment effects that could be tested for ASE are included (n=535).

Supplementary Figure 2 Classification of patterns of gene expression inheritance in ‘Cleopatra’, ‘US-897’, and ‘Flying Dragon’ in untreated (left, n=5,949) and treated (right, n=5,856) samples. **(A)** Diagram of the number of differentially expressed genes (DEGs) with a significant genotype effect in comparisons of: ‘Cleopatra’ versus ‘Flying Dragon’, ‘Cleopatra’ or ‘Flying Dragon’ versus ‘US-897’, and ‘US-897’ versus estimated mid-parent value. Solid black lines indicate comparison, with the total number of DEGs per comparison noted in the center of each line and the number of upregulated DEGs per comparison noted adjacent to each genotype. Percentages are based on the total number of tested genes (n = 20,981). **(B)** The 12 categories of gene expression inheritance were grouped into five major classifications: transgressive up in ‘US-897’, transgressive down in US-897, additive, dominant for ‘Flying Dragon’ allele, and dominant for ‘Cleopatra’ allele.

Supplementary Figure 3 NPR1 expression in ‘US-897’ and its parents. The expression of homologs of NPR1 in ‘Flying Dragon’ (n=4), ‘US-897’ (n=8), and ‘Cleopatra’ (n=8) under mock and *C*Las. NPR1 is significantly differentially expressed in ‘US-897’ compared to ‘Cleopatra’ (FDR adjusted p-value < 0.05) but not ‘Flying Dragon’.
