# Supplementary figures and images for: The genetic architecture of gene expression regulation in a Citrus x Poncirus hybrid tolerant to Huanglongbing
Source: Front Plant Sci. 2025 Sep 4;16:1627531. doi: 10.3389/fpls.2025.1627531 (PMC12443710; doi:10.3389/fpls.2025.1627531)

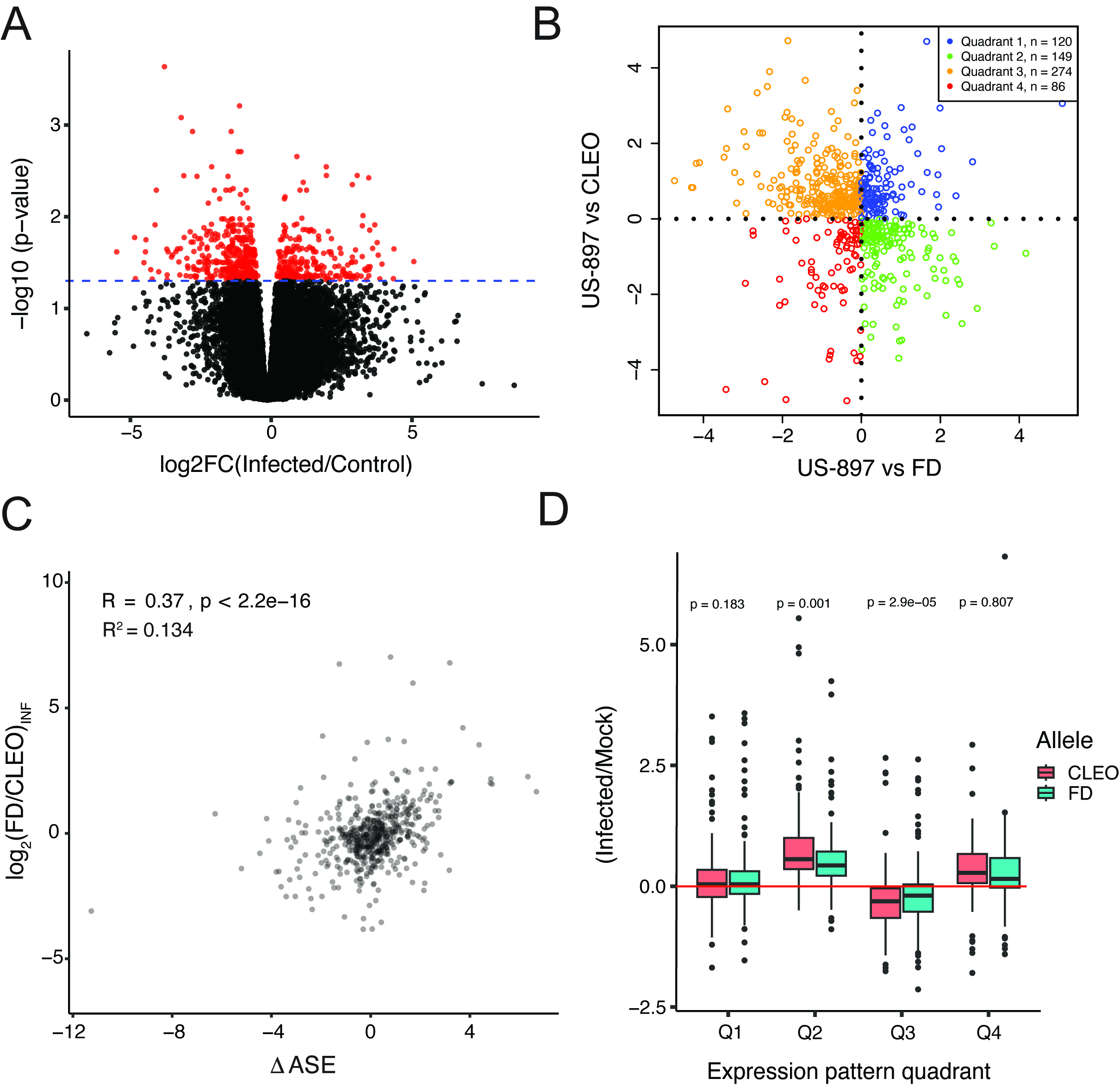

Supplement: Supplementary Figure 1 — Classification of patterns of gene expression inheritance in ‘Cleopatra’, ‘US-897’, and ‘Flying Dragon’ for genes with significant treatment effects (n=629). (A) Identification of genes with significant treatment effects. The dotted line represents the significance threshold (FDR corrected p < 0.05). (B) Categorization of gene expression patterns relative to expression in ‘US-897’ versus parental genotypes for genes with significant genotype x treatment interaction (n=629). The dot-plot compares the log2 fold-change in gene expression between infected samples of ‘US-897’ and its two parents, with points colored by quadrant. (C) The relationship between Δ ASE in US-897 (the allelic response to CLas infection) (log2(FD/CLEO)Allelic) and expression divergence between parental genotypes ‘Flying Dragon’ and ‘Cleopatra’ (log2(FD/CLEO)Parental). Only genes with significant treatment effects that could be tested for ASE are included (n=535). (D) The response of each allele in ‘US-897’ to pathogen infection for genes in each of the quadrants in (B). Only genes with significant treatment effects that could be tested for ASE are included (n=535). [file Image1.tif]

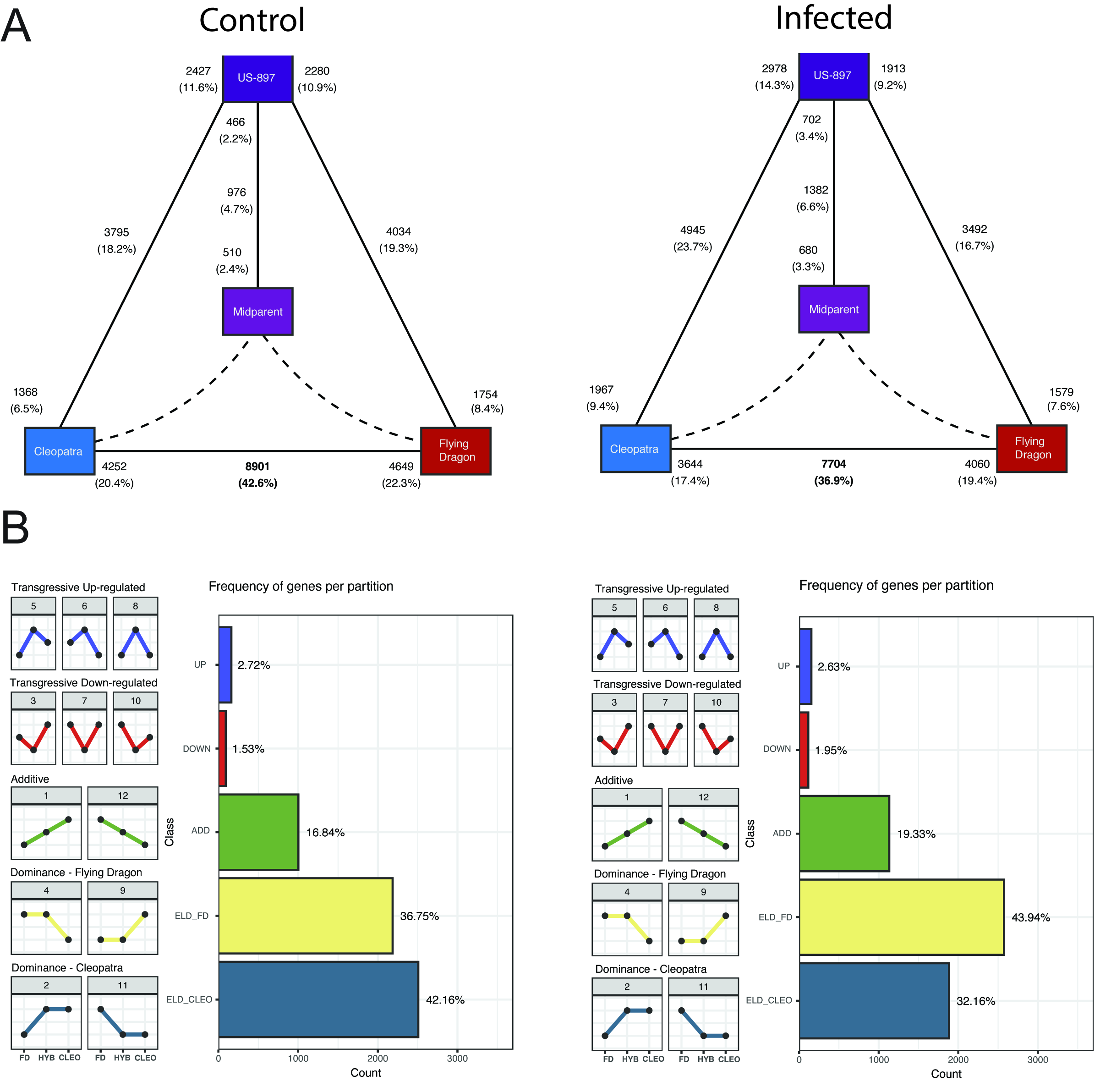

Supplement: Supplementary Figure 2 — Classification of patterns of gene expression inheritance in ‘Cleopatra’, ‘US-897’, and ‘Flying Dragon’ in untreated (left, n=5,949) and treated (right, n=5,856) samples. (A) Diagram of the number of differentially expressed genes (DEGs) with a significant genotype effect in comparisons of: ‘Cleopatra’ versus ‘Flying Dragon’, ‘Cleopatra’ or ‘Flying Dragon’ versus ‘US-897’, and ‘US-897’ versus estimated mid-parent value. Solid black lines indicate comparison, with the total number of DEGs per comparison noted in the center of each line and the number of upregulated DEGs per comparison noted adjacent to each genotype. Percentages are based on the total number of tested genes (n = 20,981). (B) The 12 categories of gene expression inheritance were grouped into five major classifications: transgressive up in ‘US-897’, transgressive down in US-897, additive, dominant for ‘Flying Dragon’ allele, and dominant for ‘Cleopatra’ allele. [file Image2.tif]

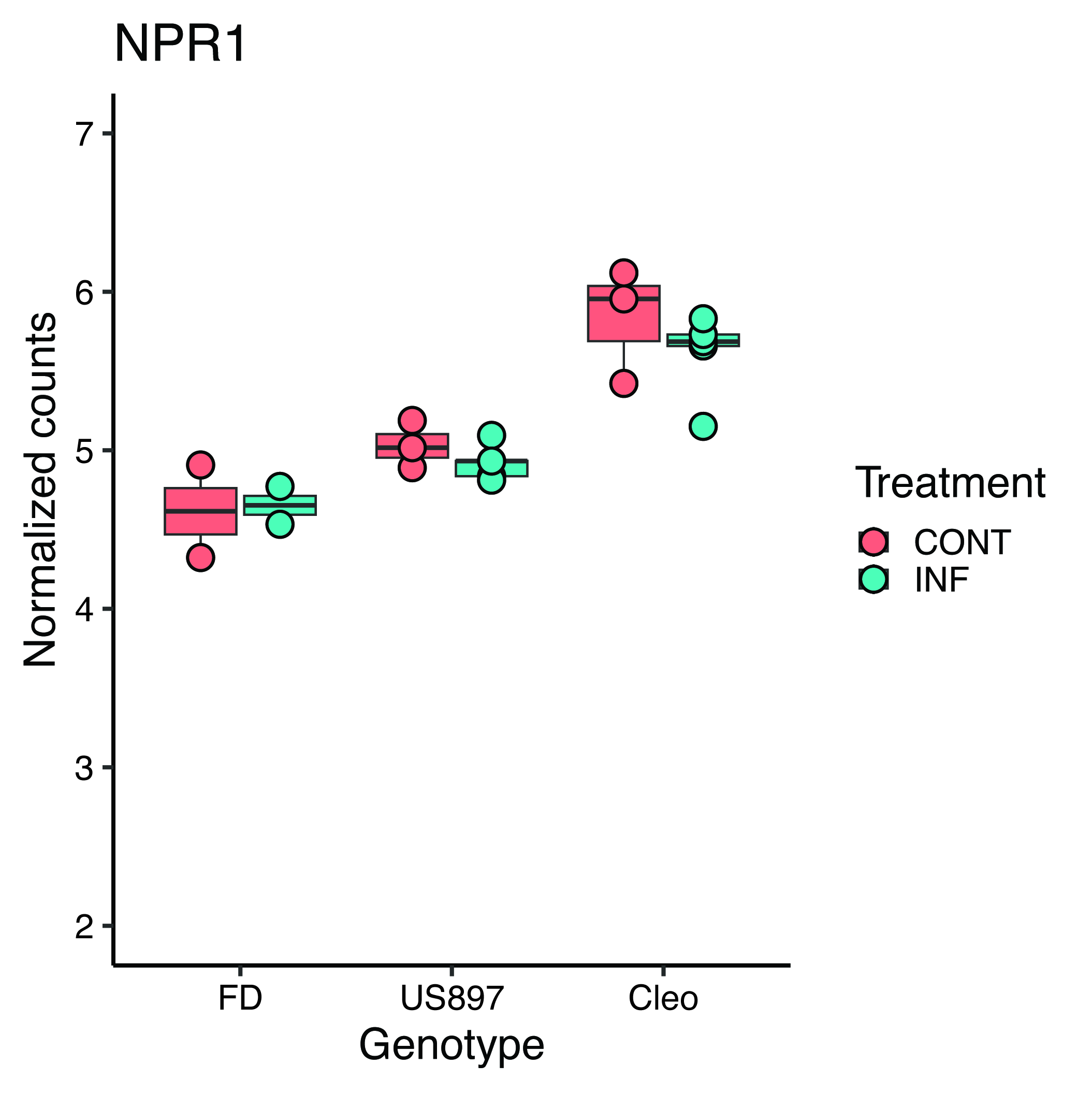

Supplement: Supplementary Figure 3 — NPR1 expression in ‘US-897’ and its parents. The expression of homologs of NPR1 in ‘Flying Dragon’ (n=4), ‘US-897’ (n=8), and ‘Cleopatra’ (n=8) under mock and CLas. NPR1 is significantly differentially expressed in ‘US-897’ compared to ‘Cleopatra’ (FDR adjusted p-value < 0.05) but not ‘Flying Dragon’. [file Image3.tif]
